# Comparisons of multiple automated anatomy‐based image‐guidance methods for patient setup before head/neck external beam radiotherapy

**DOI:** 10.1120/jacmp.v12i1.3337

**Published:** 2010-10-13

**Authors:** Nesrin Dogan, Shiyu Song, Habeeb Saleh, Jian Wu, Martin J. Murphy

**Affiliations:** ^1^ Radiation Oncology Department Virginia Commonwealth University Medical Center Richmond VA USA

**Keywords:** image registration, image‐guided setup, setup uncertainty, registration error, head/neck radiotherapy

## Abstract

The purpose was to assess the variability in automated translational head/neck setup corrections computed from several different imaging modalities and rigid registration methods using patient anatomy. Shifts were calculated using three commercial and one in‐house automated rigid registration methods for nine head/neck patients who were imaged with three different image‐guidance systems. The mean difference between the daily isocenter shifts determined by the four methods ranged from 2.8 to 12.5 mm for all of the test cases. These differences are much greater than the variability observed for a rigid imaging phantom. Image‐guided setup procedures have an uncertainty that depends on the imaging modality, the registration algorithm, the image resolution and the image content. In the absence of an absolute ground truth, the variation in the shifts calculated by several different methods provides a useful estimate of that uncertainty.

PACS number: 87,55,km, 87.57.nj, 87.59.‐e, 87.59.bd

## I. INTRODUCTION

Historically, image‐guided patient setup for external beam radiotherapy has used 2D projection images to measure the translational shifts needed for optimal beam alignment. The shifts can be determined either via 2D/2D registration to simulation images or via 2D/3D registration to the planning CT. Owing to the poor visibility of soft tissue features in 2D projections, alignment is usually established via bony landmarks or implanted fiducials. The introduction of computed tomography (CT) systems into the treatment room^(^ ^1^ ^–^ ^4^ ^)^ allows patient setup via 3D/3D image registration using bony landmarks, fiducials or soft tissue. All image‐guided setup methods provide an approximately optimal patient position with residual errors. It is often not possible to establish a ground truth to estimate those errors. When that is the case, one way to expose the uncertainty in the setup process is to acquire setup images using two or more imaging modalities, register them using two or more different methods, and then compare the calculated shifts. These tests can provide some insight into uncertainties in the setup mechanism when comparison to an absolute ground truth standard is not possible.

One can distinguish two basic sources of image‐guided setup error: (1) intrinsic instrumental uncertainties due to image resolution and rigid registration accuracy, and (2) extrinsic uncertainties that arise from the manner in which the guidance system is configured and used. Acceptance and commissioning tests for any particular guidance system measure intrinsic uncertainty using rigid phantoms that have well‐controlled imaging properties and can provide a ground truth reference. However, clinical applications introduce extrinsic uncertainties that are affected by: the imaging field of view, the anatomical region of interest that is specified, variations in image quality due to the patient's anatomy, the presence of non‐rigid structures in the field of view, and other factors that are outside the control of a phantom‐based assessment. Consequently, image‐guided setup systems that meet stringent commissioning and acceptance criteria for accuracy and precision will not necessarily deliver the same level of accuracy and precision in practice. Furthermore, two different guidance systems can identify different setup corrections for the same patient. Generally, though, there is no ground truth for actual patient setup situations and it is therefore difficult to know the actual clinical accuracy of an image‐guided setup procedure.

Intrinsic setup uncertainties have been investigated by many researchers. For example, Penney et al.^(^ ^5^ ^)^ looked at the robustness and relative accuracies of different similarity measures for 2D/3D registration. Wu and Murphy^(^ ^6^ ^)^ have shown the variability among several different 3D/3D registration algorithms applied to the same set of pelvic CT images. That study demonstrated the value of using the reversibility error (the variation in setup corrections when the source and target images are exchanged) as an indication of registration convergence and an estimate of registration accuracy in the absence of a ground truth.

The effects of extrinsic factors have received less attention. Li et al.^(^ ^7^ ^)^ have reported variations in head/neck setup corrections obtained from 2D radiography and 3D cone beam CT. The effect of respiration blurring and motion artifacts on the rigid registration of 3D CT images has been investigated by Woodford et al.^(^ ^8^ ^)^

The goal of this study was to determine the variability in setup shifts for head/neck patients due to: 1) different registration methods operating on the same image sets, and 2) different registration methods operating on different image sets of the same patient under conditions where no ground truth for the shifts was available. This amounted to a study of the extrinsic setup uncertainties in clinical applications. Each patient was imaged essentially simultaneously by three different commercial imaging systems that are presently in widespread use. Translational setup corrections were then calculated from each modality using the associated commercial software. (Rotational shifts were excluded because they are not allowable in two of the commercial registration methods.) The data for one imaging modality were independently analyzed using an in‐house rigid registration process. The imaging and registration steps were performed according to standard system operating procedures. Any one of the four results could have been used to make the patient position adjustment. Lacking a ground truth, we did not attempt to determine which of the various setup systems gave the “best” result. These tests revealed the range of setup results one can obtain in routine clinical practice, depending on what imaging modality, registration process and region of interest are used.

## II. MATERIALS AND METHODS

This study used a total of 29 daily sets of setup data for nine head/neck patients. We analyzed anywhere from one to seven daily setups for each patient. All patients were treated using the Trilogy linear accelerator (Varian Medical Systems, Palo Alto, CA) equipped with three different in‐room kV imaging systems: 1) Varian On‐Board Imaging (OBI); 2) Varian cone beam CT (CBCT); and 3) BrainLab ExacTrac (BrainLAB, Inc., Westchester, IL) X‐ray imaging. This facility allows a simultaneous comparison of multiple image‐guided setup procedures as they are used in the clinic.

Patients were immobilized with thermoplastic face masks and, where appropriate, a bite block system. Each patient had a fan beam CT (FBCT) scanned in the supine position with 3 mm slice thickness for treatment planning. At the beginning of each treatment fraction, each patient was imaged for setup using up to three different modalities: 1) a CBCT acquired with the Varian OBI system; 2) a pair of orthogonal planar radiographs taken at AP and lateral viewpoints using the Varian OBI imaging system; and 3) a pair of oblique planar radiographs taken by the BrainLab ExacTrac X‐ray imaging system. Translational setup shifts were compared for those fractions where all three types of setup images were acquired, for a total of 29 comparison tests. The X‐ray exposure settings for 2D OBI imaging were set to 100 kV, 100 mA, and 80 ms for AP and 70 kV, 100 mA, and 50 mS for lateral viewpoints. The CBCT scans were half‐fan and were acquired at standard dose exposure setting which was 125 kV, 80 mA, and 25 mS, with bowtie filter (610 projections). The ExacTrac images were acquired at H&N standard exposure setting, which was 66 kV, 100 mA and 100 mS. Typical daily imaging sessions took 2–3 minutes for OBI, 4–5 minutes (including image reconstruction) for CBCT, and 2–3 minutes for ExacTrac.

The CBCT‐FBCT shifts were calculated using both the vendor‐supplied Varian auto registration process (version 1.3) and an in‐house 3D‐3D rigid registration process. The Varian 3D‐3D registration process maximizes mutual information. The in‐house process maximizes the mean‐squared‐intensity‐difference of the two images via a steepest descent algorithm.^(^ ^6^ ^)^ Both 3D‐3D registration methods computed translations and rotations. The ExacTrac‐FBCT setup shifts were calculated by the BrainLab iterative 2D‐3D rigid registration process, which uses mutual information as the similarity measure and calculates both translations and rotations. However, because the treatment couch cannot make rotational corrections, only the translational shifts could be used.

The OBI‐FBCT shifts were obtained by registering the two OBI images to DRR simulation images calculated from the FBCT, using vendor‐supplied 2D‐2D registration software (which used mutual information and a gradient descent iterative algorithm – version 1.3). The OBI 2D/2D registration provided only translations. The automatically‐calculated OBI shifts were visually checked by the technicians to identify outlier results. If the automatic result was visually unconvincing, the registration was redone by the technicians manually. There was no clinical record of when these manual registrations were done. Therefore the OBI data contain an unknown mixture of automatic and manual registration results.

The setup results reported here were obtained using the commercial image guidance tools in typical clinical fashion according to vendor instructions. The commercial registration processes used the entire field of view and image content (i.e., no ROIs were selected and no anatomical features were highlighted). None of the images were retrospectively re‐analyzed using the commercial software. The in‐house registration process allowed the user to select a limited ROI that included either all of the image information or anatomical features selected via intensity thresholds. For the head/neck comparisons, the in‐house registration was based on bony landmarks close to the treatment site. The in‐house calculations involved retrospective analysis of the FBCT and CBCT images.

We used a CT quality assurance (QA) phantom (Catphan, The Phantom Laboratory, Salem NY) to establish a gold standard for the head/neck setup comparisons. The phantom was imaged by FBCT and then set up in the treatment room at four different positions. In the first position the phantom was placed at the nominal laser‐guided isocenter (home position); the four setup procedures then reported the shift necessary to bring it to the exact treatment isocenter. Then the phantom was moved 5 mm from the home position in the superior direction and the setup calculations were repeated. In the third trial the phantom was returned to the laser isocenter and then moved 5 mm from the home position in the anterior direction, and in the fourth trial it was moved 5 mm from the laser isocenter to the left. This test was not intended to determine the absolute setup accuracy of each method. It was intended only to compare the corrective shifts estimated by each method under idealized conditions. These would be the best results one could expect when comparing the four imaging/registration methods to one another in a clinical application.

Because different imaging systems were involved, the head/neck comparisons were made using different image content. The intent was to observe the range of setup corrections determined by the different systems and procedures under actual clinical conditions of use.

## III. RESULTS

**Figure 1:**
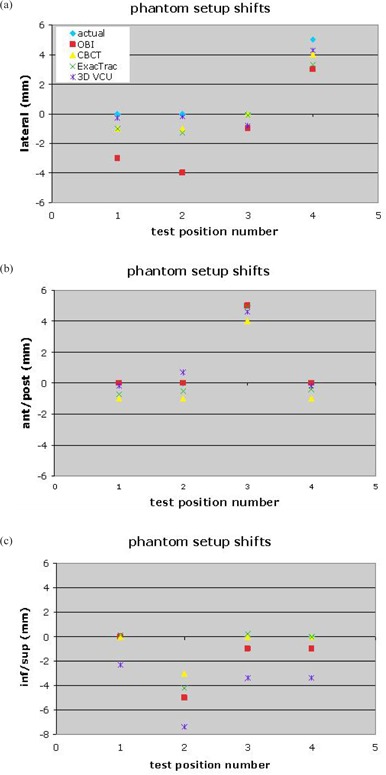
The actual shifts of a phantom relative to isocenter, compared to the setup results using: (a) the Varian OBI 2D registration; (b) the Varian 3D CBCT registration; (c) the ExacTrac 2D/3D registration; (d) the in‐house 3D CBCT registration. For test 1 the phantom was at laser isocenter; test 2 shifted the phantom 5 mm on the inferior/superior axis; test 3 shifted 5 mm on the anterior/posterior axis; test 4 shifted 5 mm on the lateral axis.

Figure 1 summarizes the positioning results for the QA phantom using the four different image‐guided setup methods. The shifts reported by the three commercial algorithms were calibrated to the treatment beam isocenter; the shifts calculated by the in‐house 3D algorithm from the OBI CBCT were not (i.e., the in‐house shifts didn't include a correction for any offset of the CBCT coordinate origin from the treatment beam isocenter). We speculate that this accounts for the systematic 3 mm difference between the in‐house 3D/3D CBCT shifts and the commercial systems' shifts in the inferior/superior direction. The variations among the individual results are the minimum uncertainties inherent in the four methods for the case of an absolutely rigid object with well‐controlled imaging properties.

Because of the potential for systematic errors in each procedure, there is no reason to believe that the four different setup measurements for a given fraction are distributed randomly about a mean value that approximates the true setup shift. Therefore we focused on the range of setup corrections that were obtained from the four different methods for each example fraction (i.e., the pairwise differences in the corrections). However, to provide the most complete picture of the results we have also compared the individual isocenter shifts for each test case with the average of the four shifts. We then computed the correlation between the shifts obtained by the different methods.

**Figure 2:**
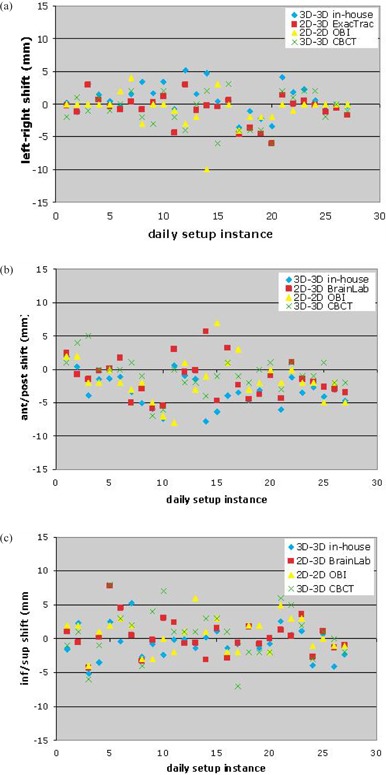
For each patient setup instance: the shifts along the (a) left‐right axis, (b) anterior‐posterior axis, and (c) inferior‐superior axis determined by the four setup calculations.

**Figure 3:**
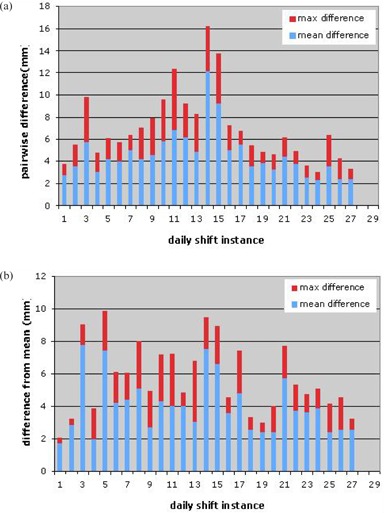
For each setup instance: the mean and maximum of (a) the pairwise Euclidean (i.e., 3D) distance between the four isocenter shifts, (b) the Euclidean distance of each of the four isocenter shifts from the average shift.

Figures 2(a)–(c) show the translational shifts measured via each method for each of the 29 cases. These results were combined to get the mean and maximum of the pairwise isocenter shift differences between the four setup measurements for each setup instance, which are shown in Fig. 3(a). In Fig. 3(b), the mean and maximum differences between each of the four estimated shifts and their average are shown for each of the 29 cases.

**Figure 4:**
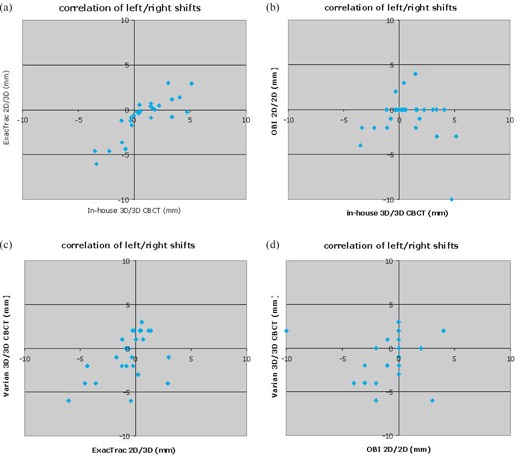
Correlation of x‐axis measurements for: (a) in‐house vs. ExacTrac (C=0.83), (b) in‐house vs. OBI (C=−0.17), (c) ExacTrac vs. CBCT (C=0.52), (d) OBI vs. CBCT (C=0.08).

For each pairwise comparison of setup results, we calculated the correlation coefficient of the shifts for each axis for the 29 cases. Table 1 shows the results. Figure 4(a) shows the plot for the most closely correlated measurements (left/right axis, in‐house 3D/3D vs. ExacTrac). Figures 4(b)–(d) show the plots for some of the less correlated measurements on the left/right axis (in‐house versus OBI, in‐house vs. CBCT, OBI vs. CBCT).

**Table 1:** Pearson's correlation coefficient between the different shift calculations for each axis.

*Axis*	*In‐house BrainLAB*	*In‐house OBI*	*In‐house CBCT*	*BrainLAB ‐ OBI*	*BrainLAB ‐ CBCT*	*OBI ‐ CBCT*
L/R	0.832	−0.174	0.451	0.219	0.524	0.084
A/P	0.453	0.152	0.582	0.161	0.316	0.334
I/S	0.507	0.536	0.510	0.460	0.621	0.505

In−house= in‐house 3D‐3D image registration method in registering Varian cone beam CT with treatment planning fan‐beam CT; BrainLAB= BrainLAB ExacTrac x‐ray imaging and 2D‐3D registration system; OBI= Varian On‐Board Imaging and Varian 2D‐2D registration system; CBCT= cone beam CT acquired with the Varian OBI system and Varian 3D‐3D registration system.

## IV. DISCUSSION

In a companion study,^(^ ^6^ ^)^ we analyzed the intrinsic precision of our in‐house 3D‐3D registration process by comparing its results to several other 3D‐3D rigid registration algorithms when all were presented with exactly the same images of the pelvis. Each algorithm focused on the same skeletal landmarks that could be assumed to be perfectly rigid. Under those conditions we estimated the intrinsic precision of the algorithms themselves to be 1–2 mm per axis.

The head/neck registration results in the present study reveal the additional setup uncertainties introduced by image modality and content, potential deformations, and by the differences between 2D/2D, 2D/3D, and 3D/3D registration procedures. The gold standard phantom tests provided an idealized situation for comparing the four different imaging modalities and registration methods. Calculated phantom positions varied by 1–4 mm.

Even though the three setup systems had been validated at the time of installation by standard commissioning procedures using calibration phantoms, the shifts they reported in our phantom measurements varied by more than one would expect (and more than what should be acceptable imprecision.) We suggest that other institutions with the ability to use multiple imaging setup systems should make a similar relative comparison as a quality assurance check. Furthermore, the alignments obtained with automated registration algorithms should always be reviewed by a radiation oncologist (not necessarily at treatment, except for the first fraction, but within a short period of time) before the shifts are applied to the patient.

The actual patient positioning results varied considerably more than the phantom results. As Table 1 and Fig. 4 show, the patient results were not correlated in any significant way and showed no clear evidence for which (if any) was the most reliable setup method. The wide range of results is probably due to variable fields of view that contain different combinations of anatomical elements. If those elements are articulated or deformed, then the images will not be accurately related by a simple rigid transformation. All of our test cases imaged the base of the skull and the neck, which is likely to present exceptional problems for rigid registration because the skeletal structure is not truly rigid. This error source has also been demonstrated by Woodford et al.^(^ ^8^ ^)^ for motion artifacts in CT images but would likely be worse if there is relative motion of high contrast bony features. Our results underscore the importance of selecting a small region of interest near head and neck treatment sites.

We also note that in the presence of rotations, a rigid registration process that has only translational degrees of freedom can give different results than a registration process that includes rotations as well.^(^ ^9^ ^)^ In our tests, the 3D‐3D CBCT and Brainlab ExacTrac registrations included rotations, while the 2D‐2D OBI registrations did not.

We have been careful to distinguish registration precision from accuracy. Precision is the variation of registration results around the similarity minimum; accuracy is the proximity of the similarity minimum to the true rigid transform. This is a particularly vexing distinction in actual clinical cases because the anatomy is often not rigid (not even the bony landmarks if the images include articulated elements). Consequently, there might not even be a true rigid transform, in which case the only meaningful error estimate is the precision. Registration precision in clinical cases can be estimated either by analyzing the behavior of the similarity surface near its minimum to estimate variability or by making multiple image‐guided setup measurements; registration accuracy in clinical cases that don't have an independent ground truth is essentially indeterminate.

## V. CONCLUSIONS

We have found that, in routine clinical procedures, the discrepancies among different registration algorithms applied via different imaging modalities to a specific setup case can often exceed what would be acceptable bounds for an image‐guided setup system's intrinsic precision. This demonstrates that phantom‐based tests and evaluations underestimate (sometimes by a large amount) the practical precision that can be obtained in a clinical application. The large variability that we observed was not necessarily due to poor performance of any particular imaging/registration method. The general loss of precision was likely due to the presence of nonrigid anatomical elements, and the way the targeted anatomy was selected and framed for each imaging modality prior to registration, rather than to technical details of the imaging and registration process. It will therefore vary depending on the anatomical site to be aligned.

The setup corrections reported here were obtained from commercially available hardware and software systems, each commissioned and used according to standard procedures. All the systems presumably met industry standards for intrinsic accuracy and precision. No particular result could be said to be demonstrably better than the alternatives. Any one of them could have been used to make patient adjustments prior to treatment. The variability among the corrections exposes extrinsic uncertainties that arise from differences in the configuration and application of the image‐guidance procedure, including field of view and imaging degrees of freedom. These extrinsic uncertainties are not small compared to interfraction patient position variability and, consequently, must be part of any PTV margin formula.

## ACKNOWLEDGEMENTS

This work was supported by NIH grants P01CA116602 and R01CA123299.
